# Unravelling the Differential Host Immuno-Inflammatory Responses to *Staphylococcus aureus* and *Escherichia coli* Infections in Sepsis

**DOI:** 10.3390/vaccines10101648

**Published:** 2022-10-01

**Authors:** Ena Gupta, Sanni Kumar, Vijay Kumar Srivastava, Juhi Saxena, Arif Jamal Siddiqui, Sudhir Mehta, Sanket Kaushik, Anupam Jyoti

**Affiliations:** 1 Amity Institute of Biotechnology, Amity University Rajasthan, Amity Education Valley, Kant Kalwar, NH-11C, Jaipur-Delhi Highway, Jaipur 303002, Rajasthan, India; 2 Department of Biotechnology, University Institute of Biotechnology, Chandigarh University, S.A.S Nagar 140413, Punjab, India; 3 Department of Biology, College of Science, University of Ha’il, Ha’il P.O. Box 2440, Saudi Arabia; 4 Department of Geriatric Medicine, SMS Medical College & Attached Hospitals, J.L.N. Marg, Jaipur 302004, Rajasthan, India

**Keywords:** Gram-negative, gram-positive, bacterial infections, host inflammatory response, NETs, iNOS, total nitrite content, pro-inflammatory cytokine

## Abstract

Previous reports from our lab have documented dysregulated host inflammatory reactions in response to bacterial infections in sepsis. Both Gram-negative bacteria (GNB) and Gram-positive bacteria (GPB) play a significant role in the development and progression of sepsis by releasing several virulence factors. During sepsis, host cells produce a range of inflammatory responses including inducible nitric oxide synthase (iNOS) expression, nitrite generation, neutrophil extracellular traps (NETs) release, and pro-inflammatory cytokines production. The current study was conducted to discern the differences in host inflammatory reactions in response to both *Escherichia coli* and *Staphylococcus aureus* along with the organ dysfunction parameters in patients of sepsis. We examined 60 ICU sepsis patients identified based on the Acute Physiology and Chronic Health Evaluation II (APACHE II) and Sequential Organ Failure Assessment (SOFA II) scores. Pathogen identification was carried out using culture-based methods and gene-specific primers by real-time polymerase chain reaction (RT-PCR). Samples of blood from healthy volunteers were spiked with *E. coli* (GNB) and *S. aureus* (GPB). The incidence of NETs formation, iNOS expression, total nitrite content, and pro-inflammatory cytokine level was estimated. Prevalence of *E. coli*, *A. baumannii* (both GNB), *S. aureus*, and *Enterococcus faecalis* (both GPB) was found in sepsis patients. Augmented levels of inflammatory mediators including *iNOS* expression, total nitrite, the incidence of NETs, and proinflammatory cytokines, during spiking, were found in response to *S. aureus* infections in comparison with *E. coli* infections. These inflammatory mediators were found to be positively correlated with organ dysfunction in both GN and GP infections in sepsis patients. Augmented host inflammatory response was generated in *S. aureus* infections as compared with *E. coli*.

## 1. Introduction

Sepsis is an acute, often fatal syndrome that significantly contributes to fatality in the critical care unit and requires early diagnosis along with proper treatment. An inflammatory condition characterized by a dysregulated immune response to infection, sepsis is considered a life-threatening organ malfunction [1]. Gram-negative bacteria (GNB) and gram-positive bacteria (GPB) that have caused underlying infections both affect the severity of sepsis. Since inflammation is the leading cause of the pathogenesis of sepsis, both GNB and GPB conspire to produce different pathologies in this disease [2]. A range of virulence factors are triggered by these pathogens, empowering them to escape the immune defense and propagate to distant organs, releasing certain toxins interacting with host immune cells with particular receptors on the cell surface and producing a poor immune response [3]. The most prevalent bacteria causing sepsis are *Staphylococcus aureus*, *Enterococcus faecalis*, *Escherichia coli*, and *Pseudomonas aeruginosa* [4].

During the common course of infection, both GPB and GNB bacteria trigger specific signaling pathways [5]. The pathogenicity of GNB is often correlated with some particular components of their membrane, particularly the lipopolysaccharide (LPS), which binds to LPS-binding protein (LBP), enlisting LPS to CD14 [6]. Toll-like receptor-4 (TLR-4) then triggers the signaling pathways of mitogen-activated protein kinase and nuclear factor κB (NF-κB) while communicating with the CD14-LPS complex [7,8]. Similarly, GPB possesses peptidoglycans (PG) and lipoteichoic acids (LTA) as virulence factors that can bind to nucleotide-binding oligomerization domain-containing proteins (NODs), which activate the TLR-2 that recognizes κ-D-glutamyl-meso-diaminopimelic acid, which subsequently activates another signaling pathway by activating NF-κB to induce pro-inflammatory cytokine production [9]. Despite the difference in clinical manifestations of gram-positive (GP) and gram-negative (GN) sepsis, similar therapeutic approaches are used in clinics to treat both pathogens.

Bacterial pore-forming toxins evoke pathophysiologic reactions, leading to differential host immune responses failing in multiple organs. As the first line of defense for the immune system against infection, neutrophils are believed to neutralize pathogens. During such a reaction, neutrophils release granular proteins and chromatin, forming extracellular fibril matrices known as NETs through an active process [10]. During conventional degranulation, cytokines are produced by the rapid secretion of interleukin-4 (IL-4) and tumor necrosis factor α (TNF-α) during exocytosis, resulting in a cytokine storm [11,12,13,14]. In macrophages, increased levels of interferon-γ (IFN-γ) promote the production of iNOS [15]. Excessive synthesis of inflammatory mediators causes the formation of reactive oxygen and nitrogen species, such as superoxide anion (O_2_.^-^) and nitric oxide (NO), resulting in the injury of tissues with increased inflammatory response. The function of oxidative stress [16], nitrosative stress [17], and hyper NETs production [18] in multiple organ dysfunctions during sepsis has been documented in previous papers from our lab. Due to the difference in induction of signaling pathways by GNB and GPB, the amount of inflammatory response would be different [1].

The current study was conducted to discern host inflammatory reactions in response to both *S. aureus* and *E. coli* bacterial infections. Herein, we determined the levels of inflammatory mediators including NOS expression, nitrite content, NETs release, and pro-inflammatory cytokines in *S. aureus* and *E. coli* bacteria-infected blood. Furthermore, different organ dysfunction parameters were evaluated in both *S. aureus* and *E. coli* infections in sepsis patients.

## 2. Materials and Methods

### 2.1. Patients Selection

Patients fulfilling the criteria of sepsis [19] were enlisted for the survey. Written and informed consent either from patients or their relatives, and ethical permission from SMS Hospital as well as Amity University Rajasthan, Jaipur (Reference number AUR/IEC/2019/01) was taken prior to initiation of the study. Patients were chosen based on (i) medical evidence of infection, (ii) hyperthermia (higher body temperature i.e., >38 °C) or hypothermia (lower body temperature i.e., <35 °C), (iii) tachycardia (elevated heart rate (>100 beats per minute), (iv) tachypnea (accelerated breathing i.e., >30 breaths per minute), and (v) evidence of poor organ function or perfusion within 12 h. Patients aged 80 years or more, also with (i) heart failure (class III or IV), (ii) hepatic insufficiency, (iv) immunosuppression (positive HIV, HBs Ag virus serologic result, malignancy), and (v) chronic antibiotic therapy were all excluded. Upon admittance to the ICU (Intensive care Unit), each patient’s clinical and demographic characteristics were recorded independently, including temperature (°C), heart rate, respiration rate, mean arterial pressure, SOFA (Sequential Organ Failure Assessment), APACHE II (Acute Physiology and Chronic Health Evaluation II), total bilirubin, creatinine, and PaO_2_/FiO_2_ ratio.

### 2.2. Blood Sample Collection

Serial blood specimens (10 mL of blood) were collected from the central venous line in ethylene diamine tetra acetic acid (EDTA) vials at the time of admission.

### 2.3. Blood Culture

Post blood sample collection, culture was performed using a BACTEC (BD, Heidelberg, Germany) blood culture system at 37 °C. The culture vials that showed growth within five days of culture incubation were separated from those that did not show growth. The growth of bacteria from liquid broth was subcultured on standardized and selected media, including blood agar, chocolate agar, and MacConkey agar, and the results were studied after 24–48 h of incubation. Standard microbiological procedures, such as Gram staining, colony features, and biochemical properties, such as catalase and coagulase, were used to examine the results, which were validated using standard manuals [20].

### 2.4. Bacterial Identification by RT-PCR

Identification of the most prevalent GNB (*E. coli*) and GPB (*S. aureus*) was done by real-time PCR (RT-PCR). Bacterial DNA was separated from whole blood using a standard kit (Nucleo-Spin^®^ Blood Quick Pure) as per the manufacturer’s instructions and used as a template for RT-PCR, which was done using gene-specific primers for *E. coli* (16s rRNA gene) and *S. aureus* (16S rRNA gene). DNA was amplified by PCR (Bio-Rad, Hercules, CA, USA), using Primer3 (http://frodo.wi.mit.edu/, accessed on 1 August 2022), a specialized web-based program, to design the primers. The primers used to amplify the *E. coli* 16s rRNA gene were F: 5′TGGCGCATACAAAGAGAAGC3′ and R: 5′TTTTGCAACCCACTCCCATG3′ and to amplify *S. aureus* 16S rRNA gene were F: 5′GAACCGCATGGTTCAAAAGT3′ and R: 5′CGGAAGATTCCCTACTGCTG3′ which amplified a 192 bp and 191 bp products, respectively. The iTaq Universal SYBR Green RT-PCR master mix was used for real-time PCR. Reactions of a total volume of 20 µL included 10 µL of iTaq Universal SYBR Green Supermix, 50 ng of DNA template, and 0.5 µL of primers. The three-step PCR procedure consisted of 2 min at 95 °C, 40 cycles of denaturation at 95 °C for 30 s, the ideal annealing 56.4 °C for *E. coli* and 59.4 °C for *S. aureus*, extension at 72 °C for 1 min, and a final extension at 72 °C for 2 min. To ensure the specificity of the PCR result as a single peak, a melting curve was run at the end of the PCR.

### 2.5. Isolation of Neutrophils from Blood

Platelets were removed from the blood by centrifuging at 200× *g* for 10 min to isolate neutrophils. After that, another 10 min of centrifugation at 700× *g* was performed. In a separate tube, white blood cells were collected from a buffy coat layer and dextran sedimented for 30 min at room temperature. Platelet-deficient plasma was kept at −20 °C for nitrite and cytokines measurement. The supernatant (4 mL) was transferred to 4 mL Percoll (1065/1080, same volume) and centrifuged at 700× *g* for 15 min at room temperature. The supernatant was removed, and the neutrophil-containing band was collected and resuspended in RPMI 1640 medium supplemented with bovine serum albumin (2%). Further neutrophils were washed with Hanks’ Balanced Salt Solution (HBSS) (NaCl 138 mM; KCl 2.7 mM; Na_2_HPO_4_ 8.1 mM; KH_2_PO_4_ 1.5 mM; Glucose 10 mM). The purity of the population was ascertained by Giemsa staining and viability by Trypan blue exclusion assay, with the results showing the population as 99% pure and viable.

### 2.6. Spiking

The pure culture of *E. coli* (ATCC *E. coli* 25922) and *S. aureus* (ATCC *S. aureus* 29213) have been taken from SMS Hospital, Jaipur. Cultures of *S. aureus* and *E. coli* were maintained in Luria–Bertani (LB) broth incubated overnight in 100 mL of nutritional broth at 37 °C. The bacteria were then diluted to a final concentration of 10^8^ cells per ml using absorbance at 600 nm wavelength [21]. Neutrophils were infected with both the bacteria with the multiplicity of infection 10 for 1 h [22].

### 2.7. iNOS Expression

For iNOS expression analysis, neutrophils were spiked with 200 μL of the culture of *E. coli* and *S. aureus* (McFarland 0.5) followed by incubation at 37 °C for 1 h. Total RNA from neutrophils (10^6^ cells) was isolated using a Tri reagent (Sigma, St. Louis, MI, USA). First, using a Revert Aid H Minus First Strand cDNA Synthesis Kit (Thermo Scientific, Madison, WI, USA) and oligo (dT) primer as directed by the manufacturer, 1 g of total RNA was reverse transcribed. The cDNA was transcribed with primers for iNOS (F: 5′TGTGCTCTTTGCCTGTATGC3′; R-5TTGCCAAACGTACTG GTCAC3′) and β-actin (F: 5′AACTGGAACGGTGAAGGTG3′; R: 5′CTGTGTGGACTTGGGAGAGG3) with the amplified products of 222 bp and 210 bp products, respectively. iNOS mRNA was quantified by RT- PCR (Bio-Rad, Hercules, CA, USA) using an iTaq Universal SYBR Green RT-PCR master mix and the same primers and cDNA as mentioned previously. Following PCR, amplicons were held at 70 °C for 10 s before being melted at 90 °C at a temperature rate of 0.1 °C per second for melting curve analysis. One melting curve cycle included 95°C for 15 s, 70 °C for 15 s, 95 °C for 10 s, and 40 °C for 3 min of cooling. As a single peak, this indicated the specificity of the PCR result. The experiment contained a control for all reaction components except the template. The reference gene β-actin was used for normalization. The difference in β-actin and iNOS quantification cycle values was used to determine the expression level of iNOS.

### 2.8. Total Nitrite Assay

The nitrite content in supernatant collected from neutrophils spiked with *E. coli* and *S. aureus* was determined by a Griess reagent kit (Thermo Fischer Scientific, Waltham, MA, USA) according to the instructions given in the manufacturer’s protocol.

### 2.9. NETs Release

NETs were generated following the protocol described previously, with modification [18,23,24]. A total of 10^6^ cells/mL of neutrophils isolated from healthy volunteers were adhered on poly-L-lysine coated petri plates, followed by washing with HBSS and treating with *E. coli* and *S. aureus* for 30 min. 4% Paraformaldehyde was used for 30 min to fix the cells, and stained with elastase antibody (1:250 dilutions) overnight. Cells were washed five times with HBSS and stained with antirabbit alexa fluor 647 antibody (1:5000 dilutions) for 4 h at room temperature in the dark. This was washed five times with HBSS. Sytox green was added and incubated for 15 min before being examined under a fluorescence microscope (Leica). Unstimulated cells were used as the negative control. The percentage of NETs was evaluated by counting the number of NETs formed by neutrophils out of the total number of neutrophils, as observed in a field in a fluorescence microscope [18,25].

### 2.10. Cytokines Estimation

Levels of TNF-α, IFN-γ, and IL-8 were estimated in supernatant collected from spiked neutrophils with *E. coli* and *S. aureus* using an ELISA kit as determined by the protocol of the manufacturer (BD Opt EIA, East Rutherford, New Jersey, USA). 

### 2.11. Statistical Analysis after the Estimation of Total Nitrite Assay and Cytokine

The data were analyzed with the SPSS program (SPSS Inc., Chicago, IL, USA) and reported as means + standard deviation from four independent experiments. The significance of the results was considered at *p* < 0.05.

## 3. Results

### 3.1. Detection of Pathogens by Blood Culture and RT-PCR

**Figure 1:**
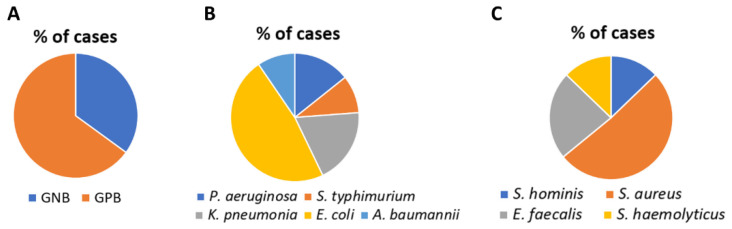
(**A**) Pie chart showing the percentage of GNB and GPB present out of the total cases. (**B**) Pie chart showing the percentage of GNB *E. coli*, *P. aeruginosa*, *A. baumannii*, *K. pneumoniae*, and *S. typhimurium*. (**C**) Pie chart showing the percentage of GPB *S. aureus*, *E. faecalis*, *S. haemolyticus*, and *S. hominis*.

The classification of Sepsis-3 is used to depict the study of population quality [21] as shown in Table 1. A total of 60 patients (average age 49.11 years; 37 males, 23 females) with sepsis were enrolled. Bacteria were detected and identified by the biochemical test. RT-PCR was done to detect the most prevalent GNB (*E. coli*) and GPB (*S. aureus*). Out of 60 cases, 39 (65%) were GP and 21 (35%) were GN (Figure 1A). Among GNB, *E. coli* (47%) *P. aeruginosa* (14%) *A. baumannii* (9%), *K. pneumoniae* (19%) and *S. Typhi* (9%) were prevalent (Figure 1B). *S. aureus* (51%) *E. faecalis* (23%), *S. haemolyticus* (12%), and *S. hominis* (12%) were predominant among GPB (Figure 1C). *E. coli* (GN) and *S. aureus* (GP) were responsible for most of the infections.

### 3.2. Expression of iNOS Generation and Estimation of Total Nitrite Content

**Figure 2:**
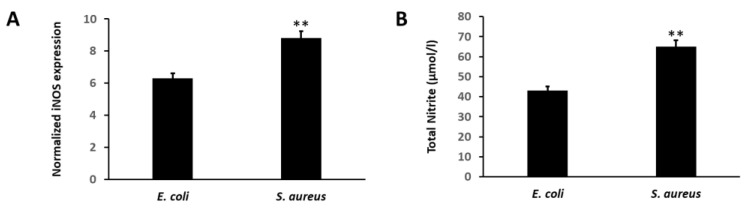
(**A**) Bar diagrams showing the RT-PCR-based relative expression of iNOS in human neutrophils spiked with *E. coli* and *S. aureus* (** *p* < 0.01). (**B**) Bar diagrams showing total nitrite content in the culture supernatant of neutrophils spiked with *E. coli* and *S. aureus* (** *p* < 0.01).

Inflammatory reactions in response to *E. coli* and *S. aureus* infection were monitored in spiked neutrophils isolated from a healthy volunteer. Augmented expression of iNOS (*p* < 0.01) was found in *S. aureus*-spiked neutrophils as compared with *E. coli*-infected neutrophils, as demonstrated by RT-PCR (Figure 2A). Nitrite, a stable product of NO generated in the supernatant due to induction of inflammatory response, is increased in *S. aureus* infection (65 ± 2.1 µ mol/L versus 43 ± 1.4 µmol/L, *p* < 0.01) (Figure 2B).

### 3.3. NETs Formation and Estimation of Cytokines

**Figure 3:**
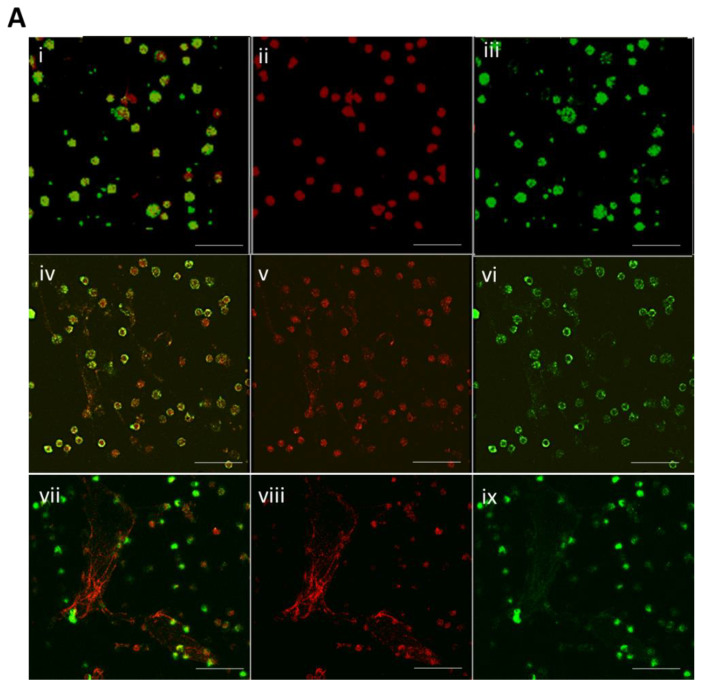

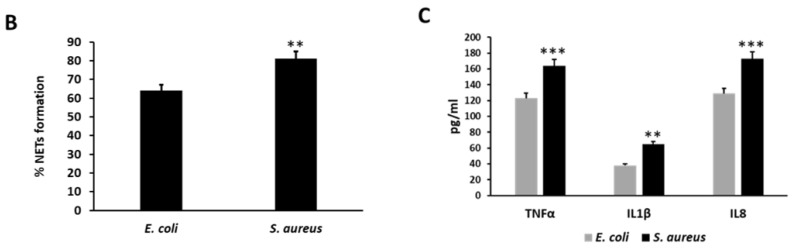
(**A**) Confocal images showing the formation of NETs in control (top panel), *E. coli* (middle panel), and *S. aureus* (bottom panel) spiked neutrophils. i, iv, and vii are merged images of neutrophils stained with SYTOX green and elastase antibody conjugated with Alexa flour 647. ii, v, and viii are the images of neutrophils stained with elastase antibody conjugated with Alexa flour 647. iii, vi, and ix are the images of neutrophils stained with SYTOX green. The scale represents 50 μm. The images are representatives of three independent experiments. (**B**) Bar diagrams representing percent incidence of NETs release measured in neutrophils spiked with *E. coli* and *S. aureus* (** *p* < 0.01). (**C**) Bar diagrams representing the level of cytokines (TNF-α, IL-1β, IL-8) in the culture supernatant of neutrophils spiked with *E. coli* and *S. aureus* (*** *p* < 0.001).

In neutrophils, the prevalence of NETs was measured and compared in *E. coli* and *S. aureus* using confocal microscopy. A greater increase in the incidence of NETs was found in response to *S. aureus* than *E. coli* (81 ± 4.2% versus 64 ± 1.7%, *p* < 0.01) (Figure 3A,B).

The cell-free content of proinflammatory cytokines was estimated using ELISA in supernatant separated from neutrophils after spiking with *S. aureus* and *E. coli*. Levels of TNF-α (164 ± 8.3 pg/mL versus 123 ± 3.4 pg/mL, *p* < 0.001), IL-1β (65 ± 3.3 pg/mL versus 38 ± 2.7 pg/mL, *p* < 0.001), IL-8 (173 ± 6.3 pg/mL versus 129 ± 8.4 pg/mL, *p* < 0.001) were found to be augmented in *S. aureus* infection as compared with *E. coli* infection (Figure 3C). The level of cytokines is less than 10 pg/mL in supernatant without bacteria presented. Our data are in accordance with a previous report which documented an almost two-fold greater amount of TNF-α generated by human monocytes in response to *S. aureus* infections than *E. coli* [26].

### 3.4. Estimation of Organ Dysfunction

**Figure 4:**
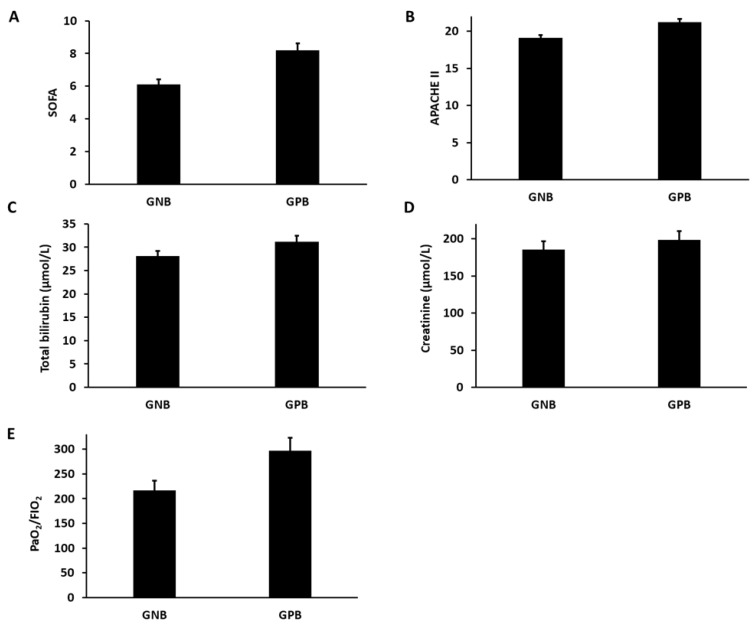
(**A**) Bar diagrams representing the SOFA score in the GPB and GNB groups (**B**) Bar diagrams representing the APACHE II score in the GPB and GNB groups (**C**) Bar diagrams representing the total bilirubin content in the GPB and GNB groups groups. (**D**) Bar diagrams representing the creatinine in the *S. aureus* and *E. coli*. (**E**) Bar diagrams representing the PaO_2_/ FIO_2_ ratio in the GPB and GNB groups.

The level of organ dysfunction parameters and organ-specific dysfunction markers were evaluated and compared in the *GPB* and GNB group of patients. We found a higher level of SOFA (8.2 ± 1.7 versus 6.1 ± 1.4) (Figure 4A) and APACHE II score (21.2 ± 3.7 versus 19.1 ± 2.9) (Figure 4B) in the GPB group in comparison with the GNB group. The total bilirubin, which assesses the hepatotoxicity, was slightly higher in the GPB group compared with the GNB group (31.2 ± 5.7µmol/L versus 28.1 ± 4.4µmol/L) (Figure 4C). The creatinine level, a potent marker of kidney function, was found to be higher in the GPB group compared with the GNB group (198.5 ± 5.3µmol/Lversus185.2 ± 6.1µmol/L) (Figure 4D). The PaO_2_/FIO_2_ ratio, which measures the lung function, was lower in the GPB group compared with the GNB group (216.4 ± 24.2µmol/L versus 296.5 ± 22.1 µmol/L) (Figure 4D).

## 4. Discussion

The purpose of this study was to investigate the detection of pathogens by RT-PCR and blood culture. We found that *S. aureus* bacteremia is a frequent clinical problem in most medical centers and is linked with high morbidity and mortality. Data showing a higher prevalence of *S. aureus* over *E. coli* are in agreement with a previous study, where 46% of isolates were GPB and 20% were GNB [27]. Similar trends were found in other reports [28], and few studies documented contrasting results with a greater occurrence of *E. coli* over *S. aureus* [29]. The possible explanation for the difference could be the design, geographic location, nature of difference of the etiological agents, and seasonal variation. *S. aureus* lives on human body surfaces and colonizes intravenous catheters, which become a source of infection in hospitalized patients, especially those with impaired immune systems, and leads to septicemia. In severe infections, *S. aureus* and *E. coli* damage internal organs including the brain, heart, lungs, as well as bones, muscles, and surgically implanted devices such as artificial joints or cardiac pacemakers [30].

We also contemplated nitrosative stress (iNOS expression /NO content/total nitrite) and pro-inflammatory cytokines (TNF-α, IFN-γ, and IL8) related to *S. aureus* and *E. coli* bacteria severity. Our PCR analysis and computational studies revealed an enhancement of iNOS in GPB (*S. aureus*) cases compared to GNB (*E. coli*). This might be related to the presence of LTA, which causes shock and multiple organ failure by activating tyrosine kinases and NF-kB in the signaling pathway, triggering the induction of iNOS [31]. In addition, macrophage-inducing iNOS is significant in the host defense system, as it stimulates NO production, which adds to bactericidal effects [32]. Similarly, LPS is required for iNOS induction, which activates certain cytokines such as IFN-γ and migration inhibitory factor that can activate macrophages to produce NO [33].

Our evidence of increased NO production adds to our understanding of the underlying cause for higher nitrite content that could be due to the presence of LTA and peptidoglycan over the cell wall of *S. aureus,* which has been shown to induce the formation of NO, followed by shock and organ injury in rats [9]. When NO, a precursor of nitrite, reacts with superoxide to form peroxynitrite, a potent oxidant, it causes cytotoxicity [34,35], thereby producing nitrous and nitric acids (NO_2_/NO_3_) in an aqueous solution, which are important signaling molecules with regulatory properties that can influence the course of inflammation and immunological regulation [36]. Our results suggest that the increased frequency of NETs is because neutrophils were already primed while spiking the blood with *S. aureus* and *E. coli*. The cell wall component of gram-positive bacteria, LTA, is a known chemo-attractant for neutrophils that helps in priming and recruitment of neutrophils at the site of inflammation [37]. Furthermore, rapid NETs release has been documented in response to *S. aureus* infection due to TLR2 and complement-mediated opsonization [38].

In sepsis, pro-inflammatory cytokines released by neutrophils and monocytes can better determine the severity of infection [39,40]. Furthermore, the correlation between increased cytokine levels in our study was conducted using a spiked neutrophil model, where the level of IFN-γ, a pro-inflammatory cytokine, was found to be upregulated in *S. aureus* septicemia as compared with *E. coli* septicemia. Contrary to our results, increased TNF-α and IL-8 were released with *E. coli* compared with *S. aureus*. The major reason behind this discrepancy could be that it includes patients with severe abdominal sepsis who harbor different pathogens involved in the immunological response [41].

We also acknowledge organ dysfunction where the above data suggest that *S. aureus* infection causes more damage to the organs as compared with *E. coli*. The possible reason could be hyperactivation of inflammatory responses including aberrant oxidative stress [16], nitrosative stress [17], and NETs release [18], which induces multiple organ dysfunction as *S. aureus* evokes more inflammatory response than *E. coli* as we found in the current study. Mechanistically, the physical interaction of iNOS and the Rac2 protein plays an important role in hyper inflammation-induced cytotoxicity in lung epithelial cells [42].

## 5. Conclusions

**Figure 5:**
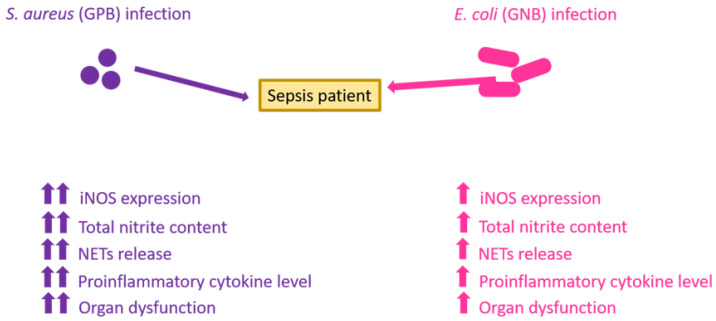
Schematic image representing increased host inflammatory responses (level of iNOS expression, total nitrite content, NETs release, proinflammatory cytokine level), and organ dysfunction in *S. aureus* (GPB) infections as compared with *E. coli* (GNB).

In summary, we found an increased prevalence of GP infections (*S. aureus*) as compared with GN (*E. coli*) during sepsis. In the whole blood spiking model, *S. aureus* evoked a greater inflammatory response (iNOS expression, total nitrite content, NETs release, and pro-inflammatory cytokine production) than *E. coli*. Thus, these inflammatory mediators have the potential to discriminate between GP and GN infections, thus bearing prognostic value. Additionally, we also found increased levels of host organ dysfunction in GPB infections than GNB infections during sepsis (Figure 5). The major limitation in our study is that data obtained from a singly type of bacteria cannot be generalized for all the GNB and GPB groups.

## Figures and Tables

**Table 1:** Patient demographic features (mean ± standard deviation).

Parameters	Value
Total number of patients	60
Age (years)	49.11 ± 12.35
Male/female ratio	37/23
Body Temperature (°C)	38.62 ± 1.28
Heart rate (beats/min)	112.45 ± 9.7
Respiratory rate (breaths/min)	27.3 ± 4.2
Mean arterial pressure (mm Hg)	86.13 ± 13.22
SOFA score	7.1 ± 1.23
APACHE II score	20.1 ± 4.1

## Data Availability

Raw data included in the manuscript is available and can be shared if required.
